# A case report of a child with severe burns treated using a multimodal approach

**DOI:** 10.3389/fped.2025.1591014

**Published:** 2025-09-29

**Authors:** Xiaoyu Liu, Guobao Huang

**Affiliations:** ^1^ School of Clinical Medicine, Shandong Second Medical University, Weifang, China; ^2^ Department of Burns and Plastic Surgery, Jinan Central Hospital, Jinan, China; ^3^ Department of Burns and Plastic Surgery, Central Hospital Affiliated to Shandong First Medical University, Jinan, China

**Keywords:** child, burns, allografts, artificial dermal scaffold, scar formation

## Abstract

**Introduction:**

Burns are a common injury in children, with severe burns carrying high disability and mortality rates.

**Methods:**

This case report summarizes the treatment, in our department, of a 9-year-old child with burn injuries caused by flame exposure, involving a total burn area of 50%, with depth ranging from third to fourth degree, and associated with mild shock.

**Results:**

Following active fluid resuscitation and anti-shock treatment, the child underwent staged wound excision and debridement, autologous and allogeneic skin grafting, and artificial dermal scaffold implantation. All wounds were successfully closed, with healing achieved within 77 days.

**Discussion:**

Insights from this case report may be applied to improve therapeutic outcomes for children with severe burns.

## 1 Introduction

Extremely severe burns in children involve more than 25% of the total body surface area (TBSA) with second-degree burns, or more than 10% TBSA with third-degree burns in children under the age of 12 years (1). Owing to their unique physiological and anatomical characteristics as well as relatively weak immune responses, children are particularly vulnerable to complications following severe burns, such as septic shock and sepsis, posing significant threat to life and challenges to treatment (2).

Excision and skin grafting in the early stages of treatment—specifically, removal of necrotic and inflammatory tissue followed by timely wound closure—reduce the risk of infection, shorten hospitalization duration, and lower mortality rates. This approach is critical for improving survival and outcomes in extensive deep burns (3). The optimal time for the initial excision of extensive deep burns is after circulatory stability is achieved, typically 3–5 days post-injury. The excision depth should be determined based on the principle of removing necrotic tissue while preserving surrounding viable tissue (3). After excising large areas of deep burns, open wounds are created, which require prompt closure and repair. Autologous split-thickness skin grafting (STSG) is the gold standard for wound coverage (4). However, autologous skin graft availability is often limited in patients with extensive burns. Combining autologous skin grafts with small cryopreserved allogeneic skin in wound repair can expand the autologous graft 9–16 times, with a graft survival rate as high as 90% (5). For deep skin and soft tissue defects, STSG may cause severe scarring and contracture, while medium- or full-thickness grafting can lead to significant donor site damage. Artificial dermis scaffolds offer a promising alternative for skin defect repair of large-area deep burn wounds with minimal trauma while restoring appearance and function (6). Furthermore, post-burn scar treatment in children is crucial, and a combination of various treatment methods is recommended for scar management and functional rehabilitation. A comprehensive, personalized long-term treatment plan should be developed through thorough evaluation (7).

Aiming to enhance treatment outcomes for children with severe burns, we report the case of a child with critical burns successfully treated with a multi-technology approach.

## 2 Methods

### 2.1 Definitions [according to the relevant consensus or guidelines of the American Burn Association (ABA) and the American Society of Critical Care Medicine]

Cutaneous burns: Injuries to the skin caused by the application of heat, cold, or caustic chemicals (8).

Infections: Suspected or proven (by positive culture, tissue stain, or polymerase chain reaction test) infection caused by any pathogen OR a clinical syndrome associated with a high probability of infection (9).

Sepsis: Life-threatening organ dysfunction caused by a dysregulated host response to infection (10).

Sepsis in children is identified by a Phoenix Sepsis Score of at least 2 points in children with suspected infection, which indicates potentially life-threatening dysfunction of the respiratory, cardiovascular, coagulation, and/or neurological systems (11).

### 2.2 Burn degree of the child

In accordance with the calculation for burn area in the Lund-Browder diagram, burn wound depth evaluation by the ABA, and burn degree classification by Li Ao Burn Science (1, 8, 12), the child was diagnosed with an extremely severe burn with TBSA of 50% and third- to fourth-degree burns.

### 2.3 Fluid resuscitation protocol

Fluid management was carried out in accordance with the Guidelines for Burn Shock Resuscitation of the ABA updated in 2023 and the Guidelines for Burn Shock Resuscitation of the ABA formulated in 2007 (13, 14). Within 24 h of admission, 3,950 ml of fluid was administered (1,950 ml in the first 8 h and 2,000 ml in the subsequent 16 h). The crystalloid-to-colloid ratio was 3:1, and 24-hour urine output was 941 ml.

### 2.4 Anti-infective treatment

The guidelines of the European Society of Intensive Care Medicine (ESICM) and the Society of Critical Care Medicine (SCCM), The Surviving sepsis campaign: international guidelines for management of sepsis and septic shock 2021 recommend immediate infusion of antibiotics for patients with suspected septic shock or high probability of sepsis (15). In the early stage of severe burns, the choice of antibiotics may be made according to the clinician's experience before the pathogen is identified. At the time of admission, the wound secretion culture results were still pending. In the meantime, we initiated empirical treatment with meropenem for a duration of 7 days.

### 2.5 Surgical treatment after fluid resuscitation

Fluid resuscitation was given; when circulation was stable, tangential excision and debridement were performed on the 4th day after admission. Considering the child's large burn area, tangential excision and debridement were performed 9 times in batches and stages. Autologous skin grafting was performed at the 1st, 5th, 6th, 7th, and 8th operation. In the 4th and 9th operations, mixed autologous and allogeneic skin grafting was performed. In the 5th procedure, artificial dermal stent implantation was performed (Supplementary Table S1).

### 2.6 Topical dressings and drugs

(1) External dressing: Biological dressing (Jiangsu Uchuang Biomedical Technology Co., LTD.; DC-ADM-c), silver ion dressing (Shenzhen AJit Medical Technology Co., LTD.; Non-self-adhesive) (2) Growth promotion drugs: recombinant human granulocyte macrophage stimulating factor (Changchun Kinsay Pharmaceutical Co., LTD.) (3) Anti-infective drugs: compound polymyxin B ointment (Zhejiang Funo Pharmaceutical Co., LTD.), silver sulfadiazine cream (Guangdong Hengjian Pharmaceutical Co., LTD.).

## 3 Case report

A 9-year-old girl suffered an accidental burn due to flame exposure. She received treatment at a local hospital, including intravenous infusion for shock, wound dressing changes, and partial decompression of the torso. She was transferred to our department for further treatment on the second day after the injury. Upon admission, the child was conscious but in a depressed mood. Assessment of vital signs indicated a temperature of 36.2°C, a heart rate of 122 beats/minute, blood pressure of 96/60 mmHg, and a respiratory rate of 20 breaths/minute. The child's weight was 20 kg.

**Figure 1:**
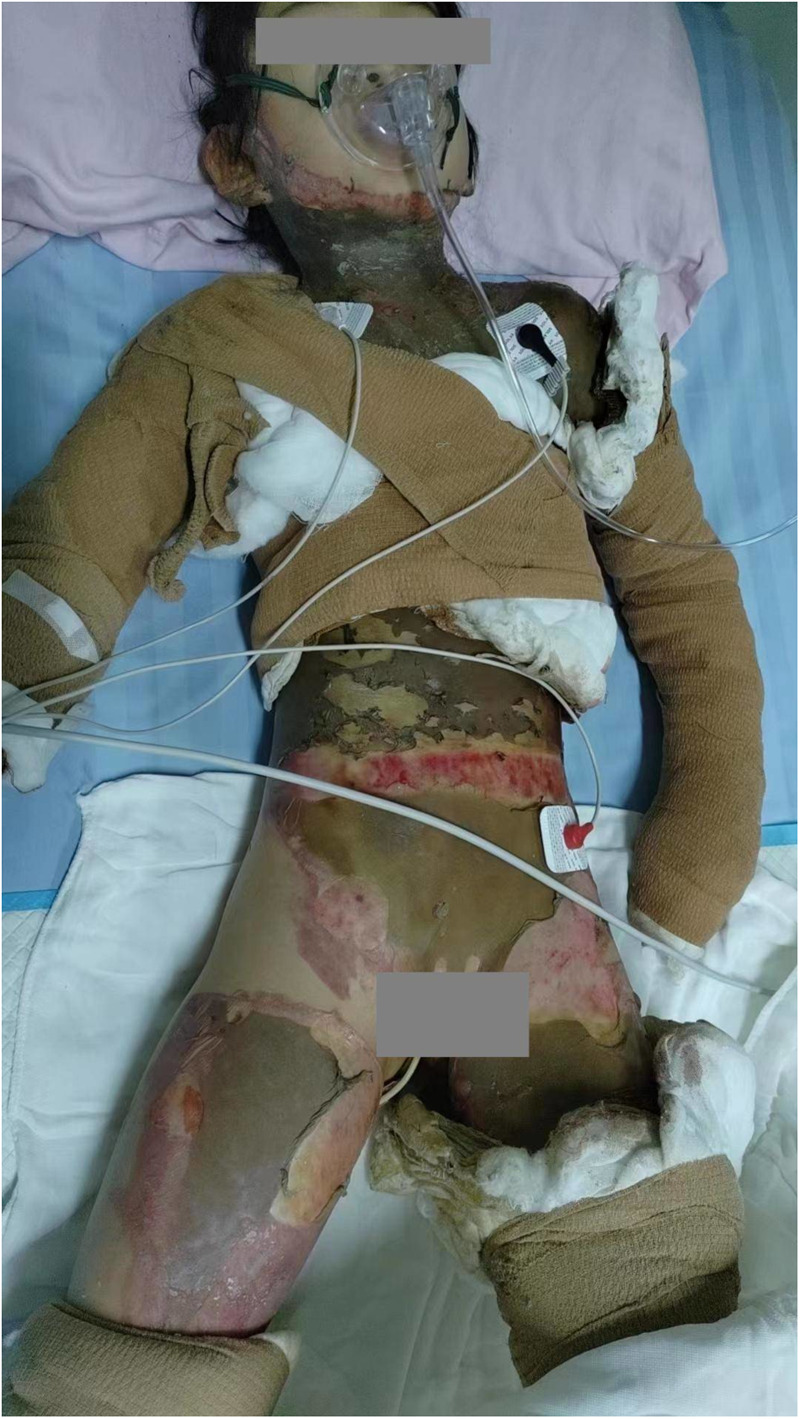
The patient had burn wounds on admission.

The child had sustained flame burns affecting multiple areas of the body, primarily the trunk, face, neck, both upper and lower limbs, perineum, and buttocks. Most burn wounds had a leather-like appearance, with areas exhibiting reduced sensation and numbness. Some wounds showed a pale base, moderate exudation, and contamination. The TBSA affected was approximately 50% (Figure 1). Laboratory tests revealed a white blood cell count of 27.51 × 10^9^/L, hemoglobin level of 13.2 g/dl, platelet count of 159 × 10^9^/L, C-reactive protein level of 40.86 mg/L, procalcitonin level of 25.71 ng/L, and albumin level of 26 g/L. Clinical diagnosis indicated multiple third-degree and fourth-degree burns caused by flame exposure—covering 50% of the TBSA—and hypovolemic shock.

Upon admission, the child received anti-infective treatment, fluid infusion, and anti-shock therapy. Once circulation stabilized, tangential excision, debridement, and biological dressing were performed in 9 sessions, following a staged approach. Tangential excision and debridement procedures involved necrotic and denatured tissue removal from the burn wound using a rotary knife until fresh bleeding was observed at the base. The wound was then irrigated alternately with benzalkonium chloride and antibiotic saline. Electrocoagulation was applied to control active bleeding. Following adequate cleansing and hemostasis, a biological dressing was applied, followed by a silver ion dressing. A mixture of growth-promoting and anti-infective drugs was then applied, and the area was pressure-bandaged with sterile dressings.

**Figure 2:**
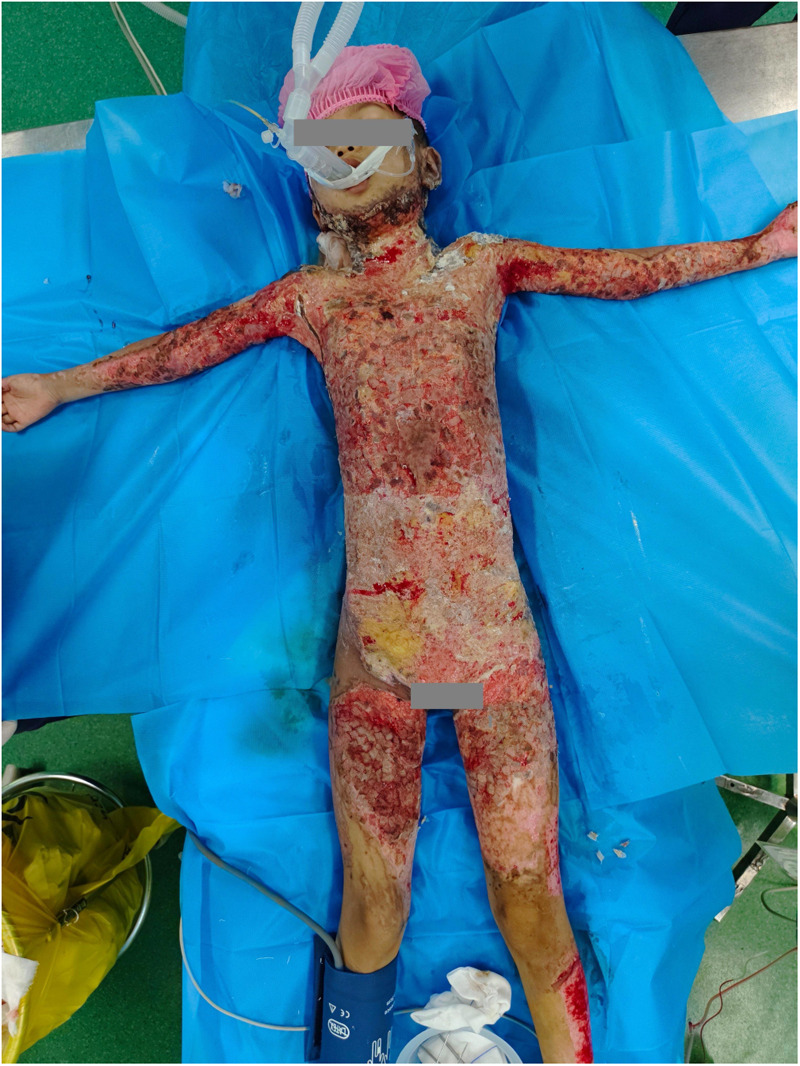
Wounds on the fifth day after allogeneic skin grafting.

At the 3rd and 10th weeks post-admission, mixed autologous and allogeneic skin grafting was performed. Allogeneic skin was harvested from both femoral regions of the child's mother, covering approximately 9% of the total skin area. The harvested allogeneic skin was then cut into 0.5 × 1.5 cm pieces. Autologous skin was obtained from the child's scalp and right lower limb, with a total area of about 8%. The autologous skin was cut into 0.5 × 0.5 cm grafts. The allogeneic grafts were placed between the autologous grafts in a neat arrangement and transplanted onto the wound site following debridement. The grafts were positioned as closely to each other as possible, minimizing gaps. A silver ion dressing was applied topically, and the wound was covered with a sterile pressure bandage (Figure 2).

**Figure 3:**
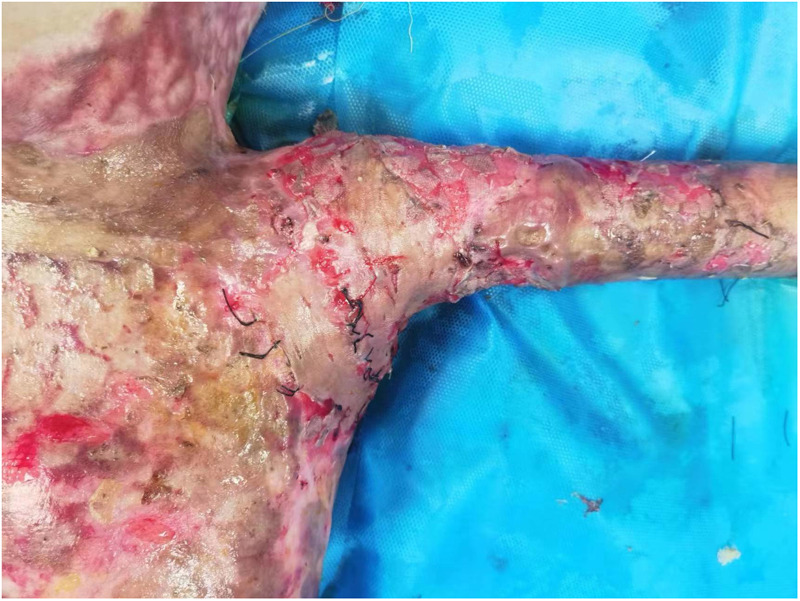
The artificial dermis scaffold in the left axilla was completely vascularized and subsequently transplanted with a large autologous skin graft.

On the 31st day following admission, shoulder debridement and artificial dermis scaffold implantation were performed. The artificial dermis scaffold was transplanted into the bilateral armpits of the child. Once the artificial dermis was fully vascularized, a large autologous skin graft was then transplanted into both armpits (Figure 3).

A rehabilitation treatment plan was developed for the child upon admission, and plasma skin regeneration therapy was administered on the 37th and 48th days of hospitalization.

**Figure 4:**
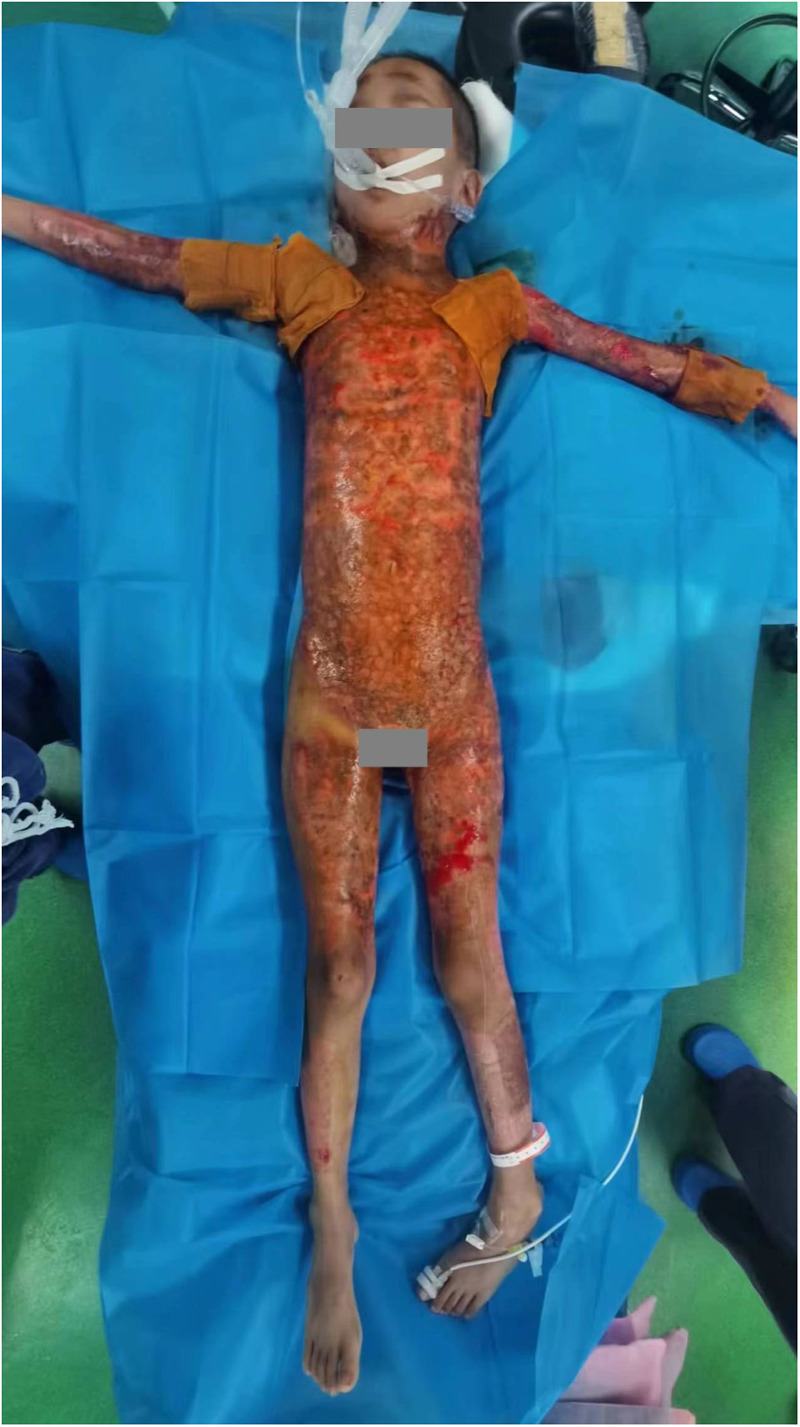
Wounds on the 77th day of admission.

The burn wound was completely closed 77 days post-burn following batch and staged surgical treatments, concluding the burn wound management (Figure 4).

## 4 Discussion

### 4.1 Fluid management in children with extremely severe burns

Hypovolemic shock can develop rapidly following burn injuries, making prompt, adequate, and controlled fluid resuscitation essential (16). Early fluid infusion significantly enhances prognosis while reducing the risk of complications (17). However, both over- and under-resuscitation can result in serious complications and increased mortality. Insufficient fluid resuscitation can lead to burn shock, poor tissue perfusion, organ failure, and necrosis. Conversely, excessive fluid resuscitation can cause fluid accumulation in tissues and interstitial spaces, leading to “fluid creep,” which can result in complications such as abdominal compartment syndrome, limb compartment syndrome, pulmonary edema, and heart failure (18). Although the 2023 American Burn Society Clinical Practice Guidelines for Burn Shock Resuscitation have been primarily designed for adults, they offer valuable insights for the treatment of children. They recommend starting resuscitation at a rate of 2 ml/kg/% TBSA—significantly lower than the traditional 4 ml formula. Here, the total volume administered in the first 24 h was 3,950 ml (approximately 2.4 ml/kg/%TBSA), with a 24-hour urine output of 941 ml (approximately 1.9 ml/kg/h). Additionally, 650 ml of fresh frozen plasma was given within the first 24 h. Therefore, in clinical practice, it is crucial to consider both the recommended fluid resuscitation formula and the individual needs of the patient in order to adjust the fluid volume appropriately.

### 4.2 Anti-infective treatment for children with severe burns

Severe burns in children often lead to septic shock and sepsis, both of which pose significant life-threatening risks. According to the International Society of Burns Injuries (ISBI), no conclusive evidence supports the early use of antibiotics in critically ill patients. However, multidrug-resistant bacterial infections complicate subsequent treatments and increases in-hospital mortality rates (19). The Guidelines for the Diagnosis and Treatment of Burn Infection (2012 edition) of the Society of Burn Surgery of the Chinese Medical Doctor Association suggest that in the early stages of severe burns, particularly when accompanied by severe shock, antibiotics may be administered based on clinical experience until pathogenic bacteria are identified. The use of potent antibiotics during the peak infection phase, particularly during edema resorption, helps better control sepsis risk (20). Thus, whether to administer antibiotics in the early stages of critical illness remains controversial. The SCCM and ESICM recommend immediate antibiotic infusion for patients with suspected septic shock or high sepsis likelihood (15). Here, the child presented with mild shock upon admission, and secretion culture results were still pending. Based on clinical guidelines and experience, the child was treated with an intravenous meropenem infusion as an anti-infective measure.

### 4.3 Wound management in children with extremely severe burns

As the primary source of risk factors and complications, wounds represent the most significant challenge for patients with burns. In severe burns, necrotic tissue should be promptly removed through batch and staged procedures, and the wound should be closed as quickly as possible (21). Prolonged wound exposure increases the risk of bacterial colonization and infection. Additionally, plasma components, including water, electrolytes, and proteins, are lost through exudation, disrupting fluid and electrolyte balance, increasing energy expenditure, and weakening immune function, which further heighten the risk of infection. In severe cases, bacteremia, sepsis, and septic multiple organ dysfunction can develop, posing life-threatening risks (22). In 1959, the severe burns treatment team at Guangci Hospital in Shanghai (Ruijin Hospital, affiliated with Shanghai Jiaotong University School of Medicine) proposed that “early eschar removal and skin grafting are crucial in reducing the risk of infection and mortality, and shortening treatment duration” (23). Upon admission to the hospital, the child underwent 9 staged operations to remove necrotic tissue, preventing the onset of complications such as bacteremia and sepsis.

To overcome the challenges of treating extensive third-degree burn wounds and treatment limitations, the Burn Center of Shanghai Ruijin Hospital has conducted comprehensive clinical research on early escharectomy, use of large allogeneic skin grafts, and mixed transplantation of autologous and allogeneic skin since 1959 (24). Hybrid transplantation combines autologous and allogeneic skin grafts, including embedding large pieces of allogeneic skin within small sections of autologous skin, using composite grafts of allogeneic skin and autologous microskin, and employing a “bricklaying” pattern of mixed autologous and allogeneic skin grafts (24–26). A large piece of allogeneic skin is often grafted onto a small piece of autologous skin—a process that is challenging to design, time-consuming, and results in significant scarring after healing. In composite transplantation, a large area of allograft is combined with autologous microskin; however, the small size of the autologous skin makes it difficult to maintain consistency during grafting. Furthermore, maintaining even spacing between the autologous skin pieces is challenging, leaving areas of the wound inadequately covered. This increases the risk of bleeding, infection, and graft failure. The demand for allogeneic skin continues to be substantial. In 2015, China prohibited the use of organs from death-row prisoners for organ transplantation, making voluntary organ donation the sole permissible source (27). This has created challenges in obtaining allogeneic skin within China.

In contrast, allogeneic skin grafting involves preparing autologous skin by cutting it into small pieces (e.g., 0.5 cm × 0.5 cm or 0.8 cm × 0.8 cm), which are uniformly transplanted onto the wound at specified intervals. Strips or stamps of allogeneic skin are placed between the transplanted skin pieces. This method has yielded positive clinical outcomes in the treatment of recurrent infections and refractory wounds.

The child weighed only 20 kg and sustained burns over 50% of their TBSA, with all burns classified as third to fourth degree. In cases of severe burns in children, extensive skin damage disrupts the natural defense barrier, significantly increasing the risk of infection and mortality (2). Therefore, wound sealing is the primary focus of treatment. However, in the present case, the child's autologous skin supply was limited and insufficient to cover the wound adequately. To compensate, a combination of autologous and allogeneic skin grafts was employed. Additionally, because the proportion of TBSA differs between children and adults, more burn wounds in children can be covered by using allogeneic skin grafts from adults with a similar TBSA (22). We obtained consent to use the patient's mother's skin as the source of allogeneic grafts. Two successful rounds of mixed allogeneic skin grafting were performed, with satisfactory clinical outcomes, and no rejection was evident in the short term. Clinically, the allogeneic skin from the mother integrated well with the child's wound. Previous studies (22, 24) have observed significant changes in the rejection pattern of allogeneic skin through careful clinical monitoring. It was noted that the allogeneic skin exhibited only repeated scaling, with the wound no longer exposed. The expanded autologous skin fused seamlessly, and the graft site was fully closed. The allogeneic epidermis was shed, while the allogeneic dermis remained beneath the newly formed autologous epidermis, enhancing wound healing (23). The temporal difference between rejection of the allogeneic dermis and epidermis is a key factor in the successful treatment of severe burn wounds using hybrid skin grafting techniques (25). Based on clinical experience and the literature, no significant rejection was observed shortly after the mixed transplantation of autologous and allogeneic skin. The potential explanations are as follows: (1) Autologous skin may induce a “skin island effect,” promoting local immune tolerance and preventing acute rejection associated with allogeneic skin transplantation. Although the precise mechanisms remain unclear, the underlying immune mechanisms may involve immature dendritic cells and keratinocytes, (28, 29). (2) The dermis is primarily composed of collagen, which has low antigenicity, allowing it to persist for extended periods without being rejected (30, 31). The allogeneic dermis, which remains intact for a prolonged period, may serve as a scaffold for the formation of new dermal tissue, thereby acting as a dermal substitute and reducing scar formation. (3) Although the immune suppression observed in children with severe burns is disadvantageous overall, it may facilitate the short-term survival of allogeneic skin grafts.

For deep skin and soft-tissue defects, simple split-thickness skin grafts are associated with reduced dermal content, significant scar hyperplasia, contracture, and poor long-term outcomes (6). In contrast, medium-thickness or full-thickness skin grafts, as well as flap transplantation, often cause greater donor site damage. Clinically, artificial dermis has been widely utilized in the treatment of deep burns to minimize scar contracture and reduce scarring at the donor site (32). The therapeutic effects have been widely recognized by clinicians worldwide (33, 34). The upper layer of the bilayer artificial dermis scaffold consists of a semi-permeable medical silicone rubber membrane, which mimics the epidermis and regulates water evaporation while preventing microbial invasion. The lower layer is a spongiform dermal scaffold composed of collagen and chondroitin sulfate, exhibiting excellent biocompatibility and low immunogenicity (35). This layer serves as a scaffold for cell growth, promoting the infiltration and growth of vascular endothelial cells and fetal bovine serum at the transplantation site, facilitating the formation of a scaffold-neovascular-cell complex. Following 2–3 weeks of full vascularization, autologous split-thickness skin can be transplanted (36). The dermal scaffold will gradually degrade and be replaced by newly formed dermal tissue (6). In this case report, the child underwent transplantation with an artificial dermal stent, followed by transplantation of autologous split-thickness skin grafts. Post-surgery, the grafts exhibited favorable survival, with minimal damage to the skin donor area. The use of artificial dermis combined with autologous split-thickness skin grafts for repairing burn wounds in children not only minimizes surgical trauma, but also reduces scar hyperplasia. Additionally, through early anti-scar treatments and rehabilitation exercises, enhanced skin durability and softness can be achieved (37).

The management of burn scars in children represents a significant challenge. Scar hyperplasia following burns negatively impacts the quality of life of affected children. Prolonged scar treatment not only imposes a substantial economic burden on families but, more importantly, subjects children to ongoing pain, disfigurement, limb deformities, and physical and mental impairments (38). Rehabilitation treatment for children with burns involves both surgical and non-surgical approaches (7). Surgical interventions are typically employed to address joint deformities caused by scar contraction, scars that significantly impair appearance, and keloids requiring surgical excision. Non-surgical treatments, in contrast, encompass a comprehensive range of methods, applied over an extended period, to achieve optimal outcomes (39, 40). Treatment should be continued until the scar reaches maturity, typically within one year (41). Key non-surgical methods include pharmacological interventions, local massage, compression therapy, orthotic use, wax therapy, medium-frequency therapy, carbon dioxide fractional lasers, dye lasers, radiation therapy, localized hormone injections, traditional Chinese medicine, and anti-tumor drug treatments (7). Plasma skin regeneration therapy has proven to be highly effective in scar treatment. This therapy does not affect skin pigmentation, thereby minimizing the risk of pigmentation changes; furthermore, it avoids epidermal vaporization, ensuring complete preservation of the separated epidermis while promoting scar healing. It is particularly beneficial for treating burn scars, significantly improving both the color and texture of the scar tissue (42). In this case, upon admission, a rehabilitation treatment plan was developed. During treatment, the joint was maintained in a functional position to the greatest extent possible, and the wound was managed with moderate pressure. Plasma skin regeneration therapy was administered on the 37th and 48th days after admission. During dressing changes, healthcare providers actively communicated with the parents, instructing them on how to perform functional exercises. This approach aimed to enhance early management and prevent excessive scar hypertrophy.

## 5 Conclusion

For children with severe burns, immediate interventions such as active fluid resuscitation, anti-shock therapy, and anti-infective treatment are essential. Necrotic tissue should be removed in a staged manner, and wound closure should be performed as soon as possible. Additionally, efforts should be made to minimize or control severe complications such as systemic inflammatory response syndrome. Hybrid transplantation of autologous and allogeneic skin can help compensate for the lack of autologous skin, reducing scar formation. The use of artificial dermal scaffolds can effectively repair deep wounds in the early stages of severe burns, promoting graft survival with minimal donor site damage. Additionally, timely anti-scar treatments and functional rehabilitation should be initiated after the burn injury.

## Data Availability

The original contributions presented in the study are included in the article/Supplementary Material, further inquiries can be directed to the corresponding author.
