# Supplementary material for: A case report of a child with severe burns treated using a multimodal approach
Source: Front Pediatr. 2025 Sep 29;13:1591014. doi: 10.3389/fped.2025.1591014 (PMC12515647; doi:10.3389/fped.2025.1591014)
Supplement: Supplementary file 1 [file Table1.docx]

Supplementary Material

# Supplementary Tables

| **Surgery Sequence** | **Surgery time (days after burn)** | **Name of the procedure** |
| --- | --- | --- |
| 1 | 4 | Left thigh skin extraction + whole body scab removal, wound expansion + autologous skin grafting of both upper limbs and trunk |
| 2 | 7 | Wound debridement and expansion + biological dressing coverage |
| 3 | 11 | Wound debridement and expansion + biological dressing coverage |
| 4 | 19 | Scalp skin extraction + double upper limb and trunk autologous skin mixed transplantation + biological dressing coverage |
| 5 | 32 | Dermatografting of scalp skin + autologous skin grafting of both upper limbs and trunk + artificial dermal scaffold implantation in both axillae + biological dressing coverage |
| 6 | 47 | Right lower extremity skin extraction + autologous skin grafting of both upper limbs and trunk + biological dressing coverage |
| 7 | 59 | Left lower limb skin extraction + autologous skin grafting of neck and right axilla + biological dressing coverage |
| 8 | 66 | Right lower extremity skin extraction + autologous skin grafting of both upper limbs and trunk + biological dressing coverage |
| 9 | 77 | Right lower limb skin extraction + bilateral upper limbs and left armpit autograft mixed transplantation + biological dressing coverage |

**Supplementary Table 1**: Details of the 9 operations performed on the child
